# UHRF1 is required for basal stem cell proliferation in response to airway injury

**DOI:** 10.1038/celldisc.2017.19

**Published:** 2017-06-13

**Authors:** Handan Xiang, Lifeng Yuan, Xia Gao, Peter B Alexander, Omar Lopez, Calvin Lau, Yi Ding, Mengyang Chong, Tao Sun, Rui Chen, Si-Qi Liu, Haiyang Wu, Ying Wan, Scott H Randell, Qi-Jing Li, Xiao-Fan Wang

**Affiliations:** 1 Department of Pharmacology and Cancer Biology, Duke University, Durham, NC, USA; 2 Department of Cell Biology, Duke University, Durham, NC, USA; 3 Department of Immunology, Duke University Medical Center, Durham, NC, USA; 4 Biomedical Analysis Center, Third Military Medical University, Chongqing, China; 5 Chongqing Key Laboratory of Cytomics, Chongqing, China; 6 Research and Treatment Center, University of North Carolina at Chapel Hill, Chapel Hill, NC, USA

**Keywords:** UHRF1, basal stem cell, proliferation, senescence

## Abstract

Cellular senescence is a cell fate characterized by an irreversible cell cycle arrest, but the molecular mechanism underlying this senescence hallmark remains poorly understood. Through an unbiased search for novel senescence regulators in airway basal cells, we discovered that the epigenetic regulator ubiquitin-like with PHD and ring finger domain-containing protein 1 (UHRF1) is critical for regulating cell cycle progression. Upon injury, basal cells in the mouse airway rapidly induce the expression of UHRF1 in order to stimulate stem cell proliferation and tissue repair. Targeted depletion of *Uhrf1* specifically in airway basal cells causes a profound defect in cell cycle progression. Consistently, cultured primary human basal cells lacking UHRF1 do not exhibit cell death or differentiation phenotypes but undergo a spontaneous program of senescence. Mechanistically, UHRF1 loss induces G1 cell cycle arrest by abrogating DNA replication factory formation as evidenced by loss of proliferating cell nuclear antigen (PCNA) puncta and an inability to enter the first cell cycle. This proliferation defect is partially mediated by the p15 pathway. Overall, our study provides the first evidence of an indispensable role of UHRF1 in somatic stem cells proliferation during the process of airway regeneration.

## Introduction

Cellular senescence represents a fundamental cell fate that has critical physiological roles in development, stem cell biology and wound healing, and pathological roles in tumorigenesis and aging [ 1,2,3, 4,5,6,7]. Thus, the study of cellular senescence at the mechanistic level could help us further elucidate the key determinants of cell fate specification and also reinforce our understanding of senescence-associated physiological and pathological conditions. Epigenetic regulation has a profound impact on many biological processes. Previous studies have implicated that epigenetic alterations occur during the senescent process and direct manipulation of epigenetic factors may change the outcome of cellular senescence [8,9,10 ]. The molecular identity of epigenetic regulators having critical roles in modulating the senescent process, however, remains largely unknown.

Human diploid fibroblasts are the most common cell type used in the senescent studies, whereas the functional importance of epithelial cell senescence is less investigated. Our lab has previously shown that epidermal growth factor receptor inhibition is sufficient to induce cellular senescence in human bronchial epithelial (HBE) cells [11], which function as basal stem cells in the airway. The conducting airways of the human lung are lined by a pseudostratified epithelium composed of luminal cells and undifferentiated basal stem cells. Immunohistochemistry analysis of airway epithelium has revealed that basal cells are cytokeratin 5 (KRT5) positive [12]. Those KRT5^+^ basal stem cells remain quiescent at steady state, but upon injury they replenish the wound by self-renewal and then give rise to early progenitor cells to further differentiate into luminal cells to reconstruct to the homeostatic state. This regeneration process is precisely controlled in a spatio-temporal manner, and disruption of the balance between basal cell proliferation and differentiation invariably results in pathological conditions, such as chronic obstructive pulmonary disease [13]. Discovery of the underlying mechanisms of epithelium regeneration by airway basal cells could shed light on the etiology of relevant respiratory diseases.

Using unbiased gene expression profiling, we found that the ubiquitin-like with PHD and ring finger domain-containing protein 1 (UHRF1) was severely reduced upon the induction of various forms of HBE cellular senescence. UHRF1 is a 90 kD multidomain protein known to bind methylated histones and hemi-methylated DNA, which also possesses ubiquitin ligase activity by virtue of its carboxyl-terminal RING finger domain [ 14,15,16]. Knockout of *Uhrf1* in mice is embryonic lethal with embryos exhibiting extreme growth retardation, and *Uhrf1*^−/−^ embryonic stem cells have pronounced DNA hypomethylation, especially in heterochromatin regions and retrotransposon elements [14]. Previous research implicates the essential functions of UHRF1 in regulating the proliferation, survival and differentiation of colonic regulatory T cells, invariant natural killer T cells and hematopoietic stem cells [ 17,18,19]. Loss of UHRF1 in neural stem cells in the developing cerebral cortex results in a postnatal neurodegeneration phenotype because of increased cell death [20]. Collectively, accumulating data suggest that UHRF1 has multiple biological functions dependent on cellular context. The function of UHRF1 in epithelial stem cell systems, however, has not been investigated.

Here, we show that UHRF1 expression is dynamically regulated during the regeneration of the mucociliary epithelium from airway basal cells *in vivo* and in three-dimensional organoid cultures. Targeted deletion of *Uhrf1* in basal stem cells results in cell cycle arrest and defective proliferation after injury without affecting cell survival or inducing premature differentiation. Importantly, UHRF1 downregulation in cultured HBE cells is sufficient to induce premature cellular senescence, and UHRF1’s capacity to suppress senescence is mainly dependent upon its ability to promote cell cycle progression. Therefore, our study comprehensively defines the function of UHRF1 in airway basal cells and the molecular mechanisms underlying UHRF1-mediated senescence suppression, with relevance to epithelial stem cell self-renewal and disease.

## Results

### UHRF1 is downregulated in several senescent contexts and UHRF1 knockdown is sufficient to induce epithelial cell senescence

**Figure 1:**
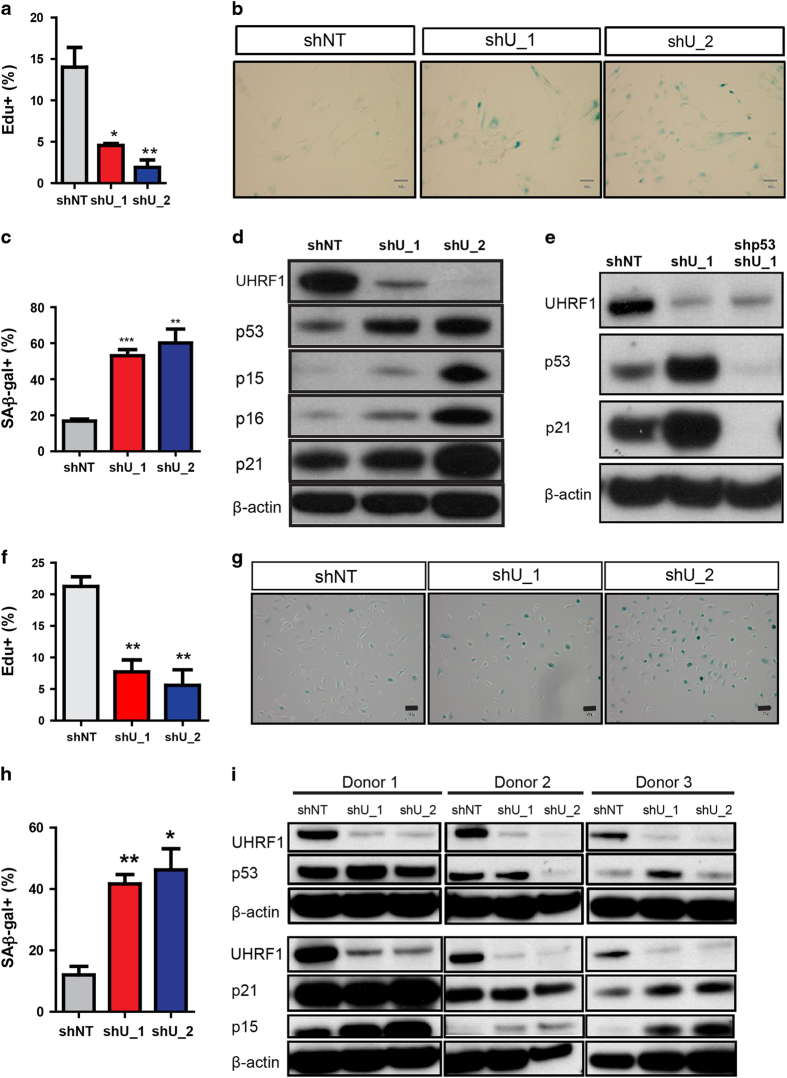
Loss of UHRF1 in IMR90 and HBE cells leads to a senescent phenotype. (**a**) Cell proliferation was measured by EdU incorporation in control (shNT) or UHRF1 knockdown IMR90 cells 6 days after virus transduction. (**b, c**) SA-β-gal staining of control and UHRF1 knockdown IMR90 cells (**b**) and quantification (**c**). (**d, e**) Whole-cell lysates from control, UHRF1 knockdown, or UHRF1 and p53 co-knockdown IMR90 cells were collected and subsequently immunoblotted with the indicated antibodies. Cells were collected 6 days after virus transduction. Note that p21 expression in UHRF1-deficient cells correlates with p53 induction. (**f**) Cell proliferation was measured by EdU incorporation in control (shNT) or UHRF1 knockdown HBE cells in culture 6 days after virus transduction. (**g, h**) Representative SA-β-gal staining is shown in **g**, and quantification is shown in **h**. (**i**) Whole-cell lysates from control (shNT) or the UHRF1 knockdown HBE cells (shU_1 and shU_2) from three independent donors were collected and subsequently immunoblotted with the indicated antibodies. Data are reported as mean±s.e.m. *P*-value was calculated based on at least three independent experiments. ***P<0.001; **P<0.01; *P<0.05.

To discover novel regulators of the senescent phenotype, we used an established model of cellular senescence comprised of sustained epidermal growth factor receptor inhibition in HBE cells [11]. Cells treated with erlotinib or dimethylsulfoxide were incubated with the fluorescent senescence-associated beta-galactosidase (SA-β-Gal) substrate C12FDG, and senescent cells were purified using flow cytometry according to the method of Debacq-Chainiaux *et al.* [21] and Yuan (in preparation). Subsequent gene expression analysis revealed significantly reduced expression of the epigenetic regulators CBX5, HELLS and UHRF1 in the senescent population compared with the non-senescent and dimethylsulfoxide controls (Supplementary Figure S1a). Quantitative real-time PCR validation confirmed that the expression of HELLS and UHRF1 was strongly repressed as early as 18 h after senescence induction, whereas CBX5 downregulation was less robust and observed only at the 48-h time point (Supplementary Figure S1a). Notably, *UHRF1* mRNA is also significantly decreased in replicative and oncogene-induced senescence based on two published gene expression data sets (GSE19864 and GSE19018). We confirmed the reduced protein expression of UHRF1 in these three senescent contexts using oncogenic H-Ras-overexpressing senescent IMR90 fibroblasts, late passage HBE cells and epidermal growth factor receptor inhibition-induced senescent HBE cells (Supplementary Figure S1b). To determine the functional significance of these findings, HELLS or UHRF1 expression was reduced using short hairpin RNA (shRNA)-mediated knockdown in HBE cells. Depletion of HELLS had no significant effect on HBE cell senescence as measured by Edu incorporation and SA-β-Gal staining (data not shown), which is consistent with previous findings in human fibroblasts [22]. In contrast, UHRF1 knockdown resulted in major impairments in cell growth (Figure 1f), mimicking the induction of cellular senescence triggered by epidermal growth factor receptor inhibition. Based on these results, we selected UHRF1 as a possible epigenetic regulator of the senescent state.

To investigate a possible role in suppressing senescence, we first used shRNA to deplete UHRF1 in IMR90 fibroblasts, a commonly used cell type in senescent studies, and observed morphological changes, cell growth arrest, and SA-β-Gal activity consistent with the induction of senescence (Figure 1a–c). Moreover, the canonical cyclin-dependent kinase inhibitors p15, p16, p21 and p53 were all upregulated as a result of UHRF1 knockdown (Figure 1d). Although UHRF1 has been previously reported to regulate the methylation status of the *Cdkn1a* distal promoter [17, 23], combinatorial targeting of UHRF1 and p53 abolished the induction of p21 (Figure 1e), indicating that p21 upregulation primarily depends on p53 status in UHRF1-deficient IMR90 cells.

We next examined the consequence of UHRF1 loss in primary HBE cells isolated from lung tissue of human donors, the cell type we used in our initial screen. As in IMR90 fibroblasts, UHRF1 knockdown in HBE cells resulted in the appearance of non-dividing, SA-β-Gal-positive senescent cells (Figure 1f–h). To examine the senescence-associated molecular alterations induced upon UHRF1 depletion, we utilized HBE cell populations obtained from three healthy human donors. Interestingly, whereas upregulation of p53 and p21 was variable among the three batches, p15 expression was uniformly increased as a consequence of UHRF1 loss (Figure 1i), suggesting that p15 is a critical regulator of UHRF1 depletion-induced senescence in HBE cells. We also tested whether UHRF1 knockdown-induced cellular senescence was due to DNA damage responses. We stained γH2AX foci in control and senescent UHRF1 knockdown HBE cells collected 6 days after UHRF1 shRNA-lentiviral infection and did not see an increase of γH2AX levels in senescent HBE cells from the UHRF1 knockdown group (Supplementary Figure S1c). Indeed, targeted knockdown of p15 was found to partially rescue the loss of proliferative capacity and increased SA-β-Gal activity phenotypes resulting from UHRF1 depletion (Supplementary Figure S2e–h), whereas downregulation of p53 could not reverse any senescence-associated phenotypes (Supplementary Figure S2a–d). The changes of p15 levels may not be due to DNA methylation, as bisulfite sequencing analysis of the *CDKN2B* core promoter showed no statistically significant changes in DNA methylation (Supplementary Figure S2i). Together, these results indicate that UHRF1 suppresses cellular senescence in multiple human cell types.

### UHRF1 is dynamically regulated and associated with basal stem cell proliferation during airway regeneration 

**Figure 2:**
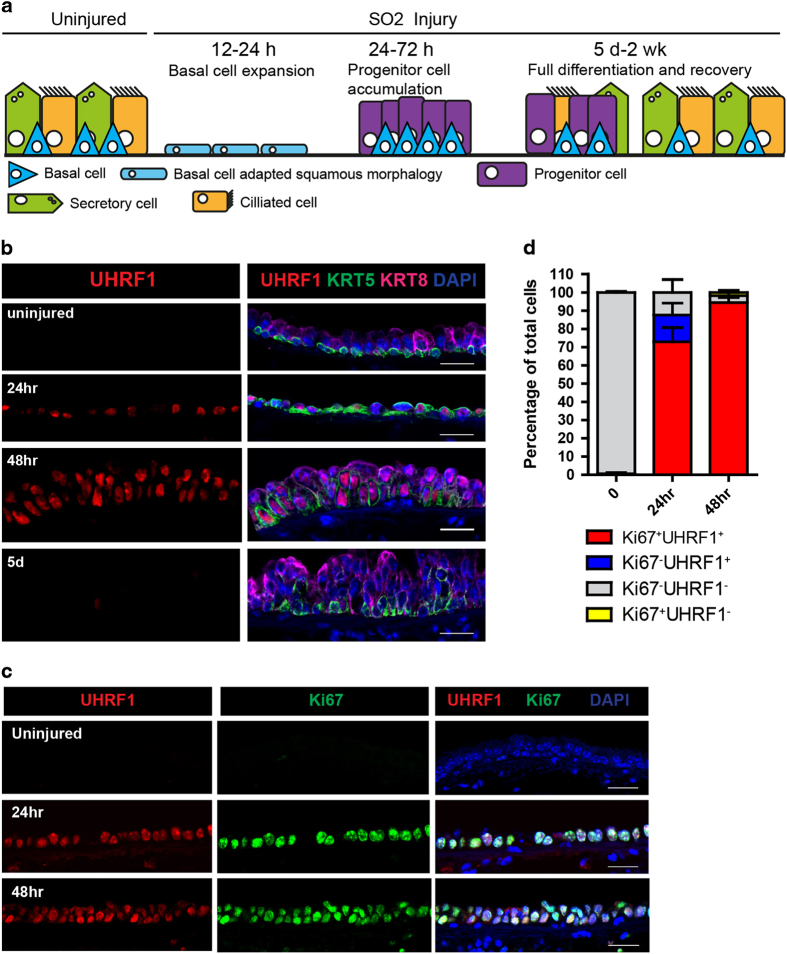
UHRF1 expression is tightly correlated with cell proliferation during airway epithelium repair. (**a**) Illustration representing the repair of the mouse tracheal epithelium by basal stem cells after SO_2_ injury. (**b**) Confocal images of mouse trachea epithelium collected at steady state, 24 h, 48 h and 5 days after SO_2_ injury. Longitudinal midline sections stained with antibodies to KRT5 (basal cell marker), KRT8 (differentiated luminal cell and early progenitor cell marker) and UHRF1. Note that UHRF1 expression was undetectable at steady state but was markedly upregulated after injury. (**c**) Confocal images of mouse trachea epithelium collected at steady state, 24 and 48 h after SO_2_ injury. Tissue sections were co-stained with UHRF1 and Ki67, a proliferation marker. (**d**) Quantification of the percentage of Ki67^+^UHRF1^+^, Ki67^+^UHRF1^−^, Ki67^−^UHRF1^+^, Ki67^−^UHRF1^−^ in total cells in the epithelium. Scale bar: 20 μm.

We next sought to determine the physiological relevance of these findings. HBE cells function as airway stem cells that can proliferate and differentiate into both secretory cells and ciliated cells in response to airway injury [12, 13]. A previous study demonstrated that *Uhrf1* is among the genes most highly upregulated in response to sulfur dioxide (SO_2_)-induced airway injury (Supplementary Figure S3), although the functional significance of UHRF1 induction for the airway repair process was not explored [24]. To investigate this, we exposed mice to 500 parts per million (p.p.m.) of SO_2_ for 4 h, which results in depletion of differentiated luminal cells expressing KRT8. These differentiated cells are replenished over the next 5 days to 2 weeks by the proliferation and differentiation of basal cells expressing KRT5 (Figure 2a). In agreement with the gene expression analysis, immunohistochemical staining revealed that UHRF1 protein was strongly induced in KRT5^+^ basal cells at 24 and 48 h after injury, which coincides with basal cell expansion, and later downregulated upon completion of the differentiation process (Figure 2b). Moreover, co-staining with Ki67 demonstrated a nearly complete coincidence of proliferating cells and UHRF1 expression (Figure 2c and d), suggesting that UHRF1 is actively involved in the process of basal cell expansion in response to airway injury.

**Figure 3:**
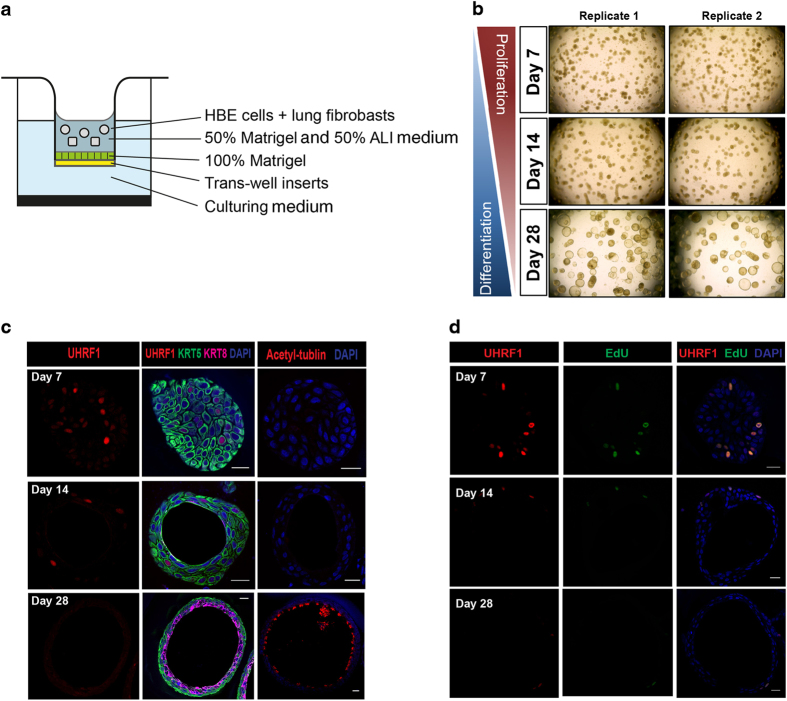
UHRF1 expression pattern in the three-dimensional tracheosphere assay. (**a**) Schematic for the three-dimensional organoid culture system. HBE cells were mixed with fibroblasts in growth factor reduced Matrigel. (**b**) Representative microscopic images of tracheosphere cultures at days 7, 14 and 28. Single basal cells proliferated and self-renewed to form visible spheres within 1 week, and spheres began to differentiate after 2 weeks. (**c**) Confocal images of individual spheres collected at indicated time points to show the expression levels of UHRF1, KRT5 and KRT8. (**d**) Confocal images of individual spheres collected at indicated differentiation time points stained with the UHRF1 antibody and the EdU click-it imaging kit. Spheres were incubated with EdU for 5 h before staining. UHRF1 was expressed in all detectable EdU-positive cells. Scale bars: 20 μm.

To test the correlation between UHRF1 expression and human airway regeneration, we took advantage of a three-dimensional tracheosphere culture system in which human basal cells are grown under conditions that favor the formation of spherical organoids containing both basal and differentiated luminal cell types [12] (Figure 3a and b). On day 7, visible spheres were already present in the culture, suggesting the rapid proliferation of single HBE cells. From 2 weeks to 1 month, the cells of each tracheosphere started to differentiate, establish apical–basal polarity and self-organize to create a stratified epithelium with luminal cells pointing toward the lumen. In this setting, UHRF1 was expressed in a subset of KRT5^+^ basal cells but excluded from KRT8^+^ luminal cells in differentiated spheres (Figure 3c), which is consistent with our earlier evidence linking UHRF1 to basal cell expansion in the damaged trachea. Importantly, EdU incorporation revealed that UHRF1 was expressed exclusively in basal cells undergoing replication during the early expansion stages of tracheosphere formation (Figure 3d).

### Loss of UHRF1 leads to irreversible cell cycle arrest and impairs proliferation of basal cells after injury

**Figure 4:**
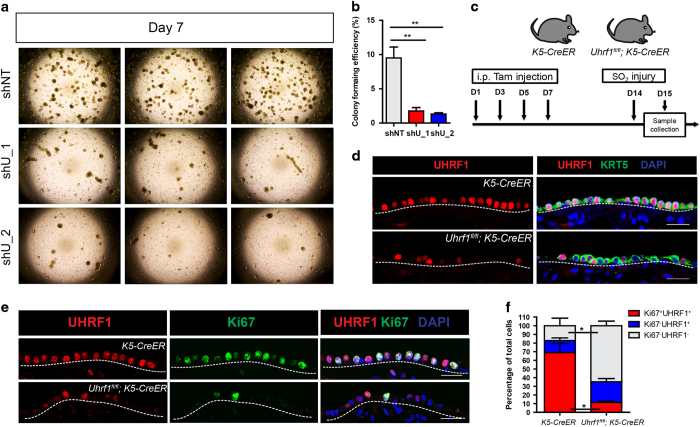
UHRF1 is required for the proliferation of basal cells in airway epithelium repair and three-dimensional tracheosphere culture. (**a**) Representative microscopic images of the tracheosphere culture from control (shNT) or UHRF1 knockdown (shU_1 and shU_2) primary HBE cells. Images were taken 7 days after seeding. (**b**) Quantification of the percentage of colony formation efficiency of each group from **a**. Data are reported as mean±s.e.m. **P<0.01, *P*-value was calculated based on three independent experiments. (**c**) Schematic of loss-of-function (*Uhrf1*^*fl/fl*^*;K5-CreER*) model. *K5-CreER* mice and *Uhrf1*^*fl/fl*^*;K5-CreER* were i.p. injected with four doses of Tmx. One week after the last injection, mice were exposed to SO_2_, and tracheas were harvested at 24 h after injury. (**d**) Representative confocal images of tracheas stained with UHRF1 and KRT5 antibodies in control (*K5-CreERT*) and loss-of-function (*Uhrf1*^*fl/fl*^*;K5-CreER*) mice. Cells in the epithelium were all KRT5^+^, indicating the success of the injury. (**e**) Representative confocal images of tracheas stained for UHRF1 and Ki67 control (*K5-CreERT*) and loss-of-function (*Uhrf1*^*fl/fl*^;*K5-CreER*) mice. The percentage of UHRF1^+^Ki67^+^ cells significantly decreased in *Uhrf1*^*fl/fl*^*;K5-CreER* mice. (**f**) Quantification of the percentage of each indicated cell population. Data are reported as mean±s.e.m. *P<0.05, three mice per group. Scale bar: 20 μm.

We next interrogated the functional ramification of UHRF1 in regulating proliferation and regeneration of HBE cells. We first knocked down UHRF1 with shRNA (shU_1 and shU_2) in early-passage HBE cells. The cells were subsequently seeded in the Matrigel for tracheosphere formation. On day 7, visible spheres could be seen in the control group (shNT), whereas UHRF1 knockdown groups derived much fewer spheres (Figure 4a and b), suggesting there was a significant decrease in the colony-forming efficiency of cells lacking UHRF1 compared with controls. Proliferation defect was mainly attributed to the abrogation of sphere formation, as UHRF1 knockdown cells in culture also had much reduced EdU incorporation rate (Figure 1f). The same finding was made with CRISPR/Cas9 genome-editing technology to knockout UHRF1: we utilized two guide RNAs to knockout UHRF1, respectively, and the tracheosphere formation capability in UHRF1-knockout cells was significantly declined (Supplementary Figure S4a). We subsequently stained those spheres formed in UHRF1-knockout group with UHRF1 antibody and found that basal cells in the spheres expressed UHRF1, indicating that spheres formed in UHRF1-knockout groups were derived from clones without successful deletion of UHRF1 (Supplementary Figure S4b).

Since a previous report has demonstrated that UHRF1 controls self-renewal versus differentiation of hematopoietic stem cells through epigenetic regulation, we examined if UHRF1 loss in HBE cells could drive premature differentiation by the air–liquid interface (ALI) culture, a well-established differentiation assay for basal cells [25]. To test this, we plated out control and UHRF1 knockdown cells and tracked two well-established differentiation markers: acetyl-tublin for ciliated cells and MUC5AC for goblet cells at early time point. We did not observe the presence of both markers in UHRF1 knockdown cells or control cells 2 weeks after seeding, although both markers were heavily upregulated in the positive controls that were well-differentiated ALI cultures from unmanipulated HBE cells (Supplementary Figure S5a). These data demonstrated that UHRF1 deficiency does not drive premature differentiation and that the impact of UHRF1 on stem cell differentiation is likely tissue specific. In addition, knockdown of UHRF1 in HBE cells did not affect cell viability indicated by Annexin V and propidium iodide (PI) staining (Supplementary Figure S5b). Treating UHRF1-deficient HBE cells with a pan-caspase inhibitor (Z-VAD-FMK) had no effect on sphere formation, suggesting apoptotic death was not the reason for the lack of sphere formation in HBE cells (Supplementary Figure S5c and d). Together, these results indicate that UHRF1 is essential for tracheosphere formation by virtue of its requirement for basal cell proliferation.

To rigorously test whether UHRF1 is necessary for basal cell expansion *in vivo*, we utilized a conditional mouse model in which CreER expression is specifically restricted to KRT5-expressing cells, allowing for inducible Cre-Lox recombination in a basal cell-specific manner. Intraperitoneal tamoxifen injection followed by SO_2_-mediated airway injury in *Uhrf11*^*fl/fl*^*;K5-CreER* mice resulted in heterogeneous UHRF1 expression in the KRT5^+^ basal cell layer 24 h after the injury (Figure 4c and d), indicative of partial gene knockout in line with previous studies [25, 26]. This procedure led to severely reduced basal cell proliferation as measured by Ki67 staining. Importantly, proliferating Ki67-positive cells were uniformly those that maintained UHRF1 expression (Figure 4e and f), suggesting that basal cells lacking UHRF1 are incapable of proliferating to repair the damaged epithelium after injury.

### UHRF1 deficiency results in a senescence-associated gene signature and genome-wide DNA hypomethylation 

**Figure 5:**
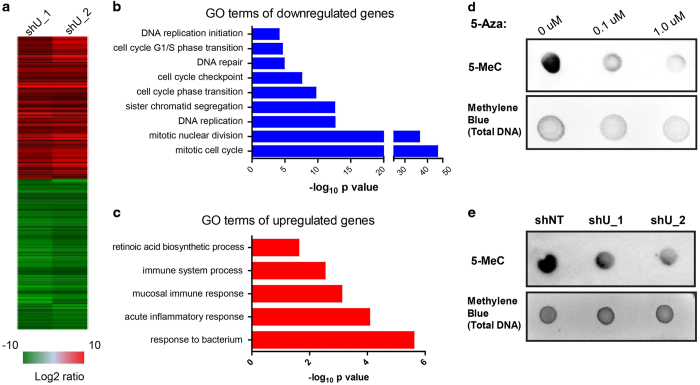
UHRF1 knockdown in HBE cells results in a senescence-associated gene expression signature and global DNA hypomethylation. (**a**) Heat map of downregulated and upregulated genes using two independent shRNAs. (**b**, **c**) Most highly represented molecular pathways altered as a result of UHRF1 depletion based on gene ontology analysis. (**d**) HeLa cells were treated with 0, 0.1 or 1 μM 5-Aza for 1 week and genomic DNA was extracted. 5-methylated cytosine (5-MeC) levels were measured by DNA dot blot assay and total DNA was stained with methylene blue. (**e**) Genomic DNA was extracted from control and UHRF1 knockdown HBE cells, and the same procedure was used to determine the ratio of 5-MeC to total DNA as indicated for each condition.

The results described above show that UHRF1 is necessary for the maintenance of HBE cell division, and this function is required for the reconstitution of the bronchial epithelium by basal stem cells after lung injury. At the molecular level, UHRF1 has been shown to coordinate histone modifications with DNA methylation, chromatin marks that ultimately function to regulate gene expression. To gain mechanistic insight into the requirement of UHRF1 for basal stem cell maintenance, we performed global gene expression analysis of HBE cells lacking UHRF1 using RNA-Seq (Figure 5a). Subsequent gene ontology analysis indicated that downregulated genes were mainly involved in cell cycle progression, with the most highly represented pathways consisting of the mitotic cell cycle, nuclear division, DNA replication and G1/S phase transition (Figure 5b). These results were unsurprising given our previous observations that UHRF1 is necessary to suppress HBE cellular senescence, but provide additional evidence that UHRF1 functions chiefly as a regulator of cell cycle progression. Interestingly, pathways upregulated as a result of UHRF1 knockdown were involved predominantly in immune and inflammatory responses (Figure 5c). This finding likely reflects the induction of a senescence-associated secretory phenotype, which is a common property of senescent cells that can have pleiotropic effects on surrounding tissue [27]. Indeed, the levels of several major cytokines known to be commonly involved in the senescence-associated secretory phenotype, including interleukin-6 and interleukin-8, were induced following the loss of UHRF1 in the RNA-Seq data (Figure 5c). Therefore, UHRF1 appears to regulate a broad program of cellular senescence consisting of both stable cell cycle arrest and the generation of a senescence-associated secretory phenotype.

The SRA domain of UHRF1 is known to preserve cytosine methylation following DNA replication via concomitant binding of hemi-methylated DNA and the maintenance methyltransferase DNMT1. A recent study reports that UHRF1 can suppress IMR90 fibroblast senescence, and senescence induced by UHRF1 knockdown is partially dependent on reduced expression of DNMT1 [28]. To examine global DNA methylation changes induced by UHRF1, we performed DNA dot blot assays using a 5-methylcytosine antibody. This assay was validated using the cytosine analog 5-azacitidine, which caused dose-dependent decreases in the abundance of 5-methylcytosine in HeLa cells (Figure 5d). Like 5-azacitidine, UHRF1 knockdown in unsynchronized HBE cells using two independent shRNAs resulted in broadly defective DNA methylation as measured by 5-methylcytosine staining (Figure 5e). However, as our RNA-seq data revealed that numerous cell cycle genes are downregulated upon UHRF1 knockdown (Figure 5b), which would not be predicted to result from reduced promoter methylation, we investigated additional mechanisms by which UHRF1 loss may impede cell cycle progression.

### UHRF1 is required for DNA replication factory formation evident by the punctate PCNA immunostaining pattern

**Figure 6:**
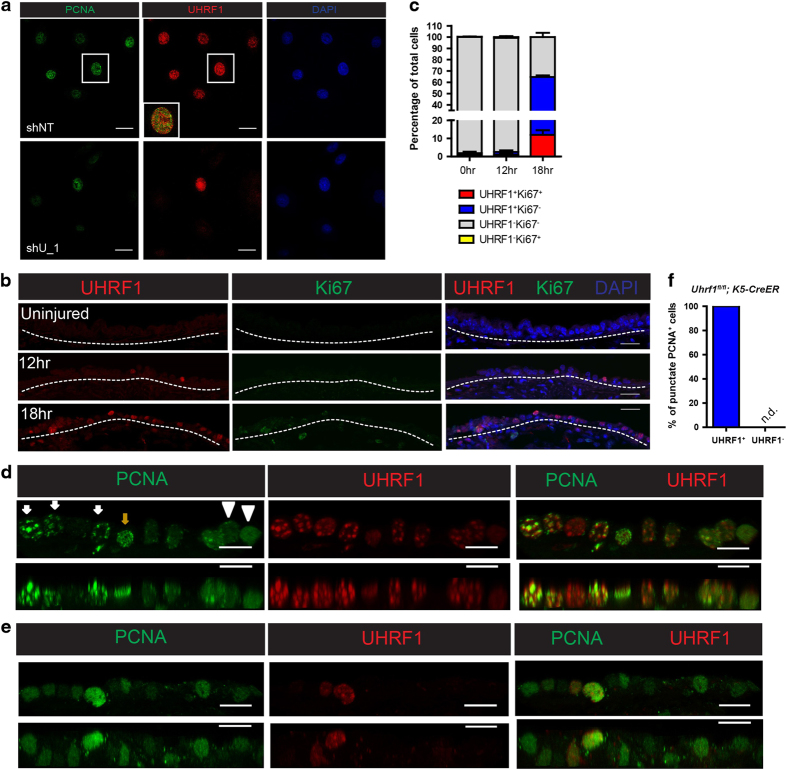
UHRF1 is prerequisite for the DNA replication factory formation. (**a**) Representative confocal images of control and UHRF1 knockdown cells co-stained with the indicated antibodies. Cells were collected 3 days after virus transduction when UHRF1 expression began to decrease. (**b**, **c**) Both UHRF1 and Ki67 expression in basal cells were examined at early time points after airway injury and quantification of cells with different UHRF1 and Ki67 expression levels were shown in **c**. These data indicate that 24-h post-injury was the time point when basal cells progressed to the S phase in the first cell cycle. Scale bar: 20 μm. (**d**, **e**) Representative confocal images of the tracheal epithelium from *K5-CreER* mice or *Uhrf1*^*fl/fl*^*;K5-CreER* mice 24 h after SO_2_ injury stained with the indicated antibodies. Top panels are projections generated from z-stack confocal images. Bottom panels are z-direction images. White arrowheads indicate cells without punctate PCNA staining patterns. Brown arrows indicate cells with punctate staining pattern in early to middle S phase. White arrows indicate cells with punctate staining pattern in late S phase. (**f**) Quantification of the percentages of UHRF1 status in punctate PCNA cells. Scale bar: 10 μm.

Given that *Uhrf1*-knockout basal cells could not proliferate in response to the injury, and the RNA-seq data indicate a cell cycle arrest phenotype, we hypothesized that UHRF1 is required for the G1 to S phase transition. To further study how UHRF1 affects this transition, we performed cell cycle progression analysis of IMR90 fibroblasts expressing a control or UHRF1-targeting shRNA. We used fibroblasts here because IMR90 cells can be easily synchronized to G1 phase by removing serum from the culture media. Fibroblasts were synchronized by serum withdrawal, and EdU incorporation and DNA content were measured after release using flow cytometry (Supplementary Figure S6a). Synchronized cells started to divide after serum release and reached peak proliferation at 24 h when most cells were advancing through the first cell cycle. We observed a robust proliferative defect at 24 h concomitantly with an increased proportion of cells in the G1 phase, indicating that UHRF1 is critical to initiate DNA replication during the first cell cycle (Supplementary Figure S6b). DNA replication factories are large protein complexes involved in DNA replication, which can be visualized by the punctate immunostaining pattern of the DNA clamp PCNA [29, 30]. Previous studies have suggested the presence of UHRF1 in the replication factory together with PCNA [14, 31], but the impact of UHRF1 on the formation of DNA replication factories to initiate DNA replication during the first cell cycle is not well understood, especially in the primary cells. To determine whether the observed G1 arrest was a result of cells’ inability to form the replication machinery in the absence of UHRF1, we performed immunocytochemical staining of UHRF1 and PCNA in proliferating HBE cells. In the early time points after UHRF1 shRNA-lentiviral infection before UHRF1 knockdown had occurred, S phase cells were observed to have overlapping, punctate staining patterns for both UHRF1 and PCNA, indicative of the presence of both proteins in a complex at the replication fork (Supplementary Figure S7a). UHRF1 and PCNA also colocalized in the nuclei of cells expressing a non-targeting shRNA. In contrast, in HBE cells lacking UHRF1 expression, PCNA immunostaining was diffuse throughout the nucleus (Figure 6a), suggesting that UHRF1 is required for the formation of DNA replication factories in order to initiate DNA synthesis.

To corroborate these findings *in vivo*, we performed immunohistochemical staining of the mouse airway using antibodies specifically recognizing UHRF1 and PCNA. Uninjured trachea had no detectable expression of either protein (Supplementary Figure S7b) as basal cells are normally quiescent. To further explore whether basal cells enter the first cell cycle at 24 h after airway injury, we examined UHRF1 and Ki67 expression at 0, 12 and 18 h after injury and found some of basal cells started to express UHRF1 but not the proliferation marker Ki67 at 18 h after injury, indicating that basal cells had exited the quiescent state and progressed to the G1 phase (Figure 6b and c). As there was still a small proportion of UHRF1^+^Ki67^−^ basal cells at 24 h (Figure 2c and d), these data suggest that the majority of basal cells undergo their first cell cycle at 24 h after injury, which is a suitable time point to assess the impact of UHRF1 deficiency on DNA replication factory formation.

In control mice at 24 h after injury, we observed punctate PCNA staining, and PCNA and UHRF1 colocalized in the nuclei of proliferating basal cells during late S phase, as indicated by large PCNA puncta (Figure 6d, white arrows). Importantly, deletion of *Uhrf1* using tamoxifen-mediated CreER activation completely abolished the punctate staining pattern of PCNA in cells lacking UHRF1 (Figure 6e and f), confirming the necessity of UHRF1 to promote the formation of DNA replication factories to activate DNA replication in response to tracheal injury. Interestingly, *Uhrf1* deletion did not affect PCNA expression in the nucleus, but inhibited active replication machinery formation as indicated by the loss of PCNA puncta (Figure 6e and f). The formation of DNA replication factories is a multi-step process involving the licensing of replication origins, assembly of a pre-initiation complex and activation of pre-initiation complexes to form functional replisomes [32]. Our data do not provide evidence to reveal which of these steps are directly impacted by UHRF1 loss. Using a chromatin fractionation assay, we have preliminary data to show that UHRF1 loss does not affect the loading of ORC2 and several MCM factors to chromatin and thus is unlikely to regulate replication origin licensing (data not shown). How UHRF1 modulates the assembly of DNA replication factories warrants further investigation.

## Discussion

In this study, we found that UHRF1 is a critical regulator of the senescent state *in vitro* and that loss of UHRF1 induces the irreversible cell cycle arrest of basal cells and impairs airway regeneration *in vivo*. Using a genetically engineered mouse model, we provide the first evidence to dissect the function of UHRF1 in airway basal stem cells. Loss-of-function experiments *in vivo* and *in vitro* suggest that UHRF1 sustains the proliferation and self-renewal of basal cells but does not control lineage choices related to differentiation or apoptotic cell death.

In previously published studies utilizing *Uhrf1* conditional knockout mouse models, UHRF1 was shown to target multiple downstream pathways resulting in diverse biological functions. For example, UHRF1 suppresses *Cdkn1a* expression through DNA methylation to facilitate the expansion of colonic regulatory T cells. In invariant natural killer T cells, UHRF1 positively regulates the Akt–mammalian target of rapamycin (mTOR) pathway to promote survival and effector differentiation, possibly independent of its DNA methylation function, whereas UHRF1 controls the cell fate specification of hematopoietic stem cells and protects them from erythroid-biased differentiation by epigenetically suppressing the expression of several differentiation genes. Here, we found that UHRF1 regulates the regeneration of airway basal cells by promoting proliferation after airway injury, suggesting that the functions of UHRF1 are largely cell type specific.

There are several possible explanations for these distinct roles of UHRF1 in different cell types. One possibility is that UHRF1 has tissue-specific targets, which could be revealed by genome-wide chromatin immunoprecipitation sequencing or RNA sequencing. This approach would further help to address the functional differences between UHRF1 and DNMT1, as UHRF1 has been reported to act as a transcriptional activator independent of its role in DNA methylation [33, 34]. Second, we speculate that intrinsic differences in cell cycle regulatory mechanisms are likely to result in discrete UHRF1 functions in the control of cell proliferation. Murine embryonic stem cells can proliferate indefinitely and lack the cell cycle-dependent Cdk/cyclin activity that is associated with the G1 to S transition in normal somatic cells [35]. *Uhrf1*^−/−^ embryonic stem cells have been reported to proliferate normally [14], whereas *Uhrf1*^−/−^ airway basal cells arrest in the G1 phase and cannot progress to the S phase, indicating that UHRF1 may affect the activity of cyclin-dependent kinases controlling the G1/S transition partially through the p15 pathway in basal cells. Additional mechanisms may also exist to prevent *Uhrf1*^−/−^ cells from entering the G1/S transition so that active replication complexes cannot form to initiate DNA replication. We did not observe a DNA damage response triggered by DNA replication stress, which is consistent with the lack of active replisomes in UHRF1-deficient cells. Detailed biochemical studies are required to determine the precise mechanism by which UHRF1 loss triggers the G1/S checkpoint.

A future avenue of investigation concerns the molecular mechanism by which UHRF1 is downregulated in senescent cells. Previous studies on *UHRF1* gene regulation focused mainly on the mechanisms underlying cell cycle-dependent control of UHRF1 expression. The transcription factor E2F1 has been reported to bind to the first intron of *UHRF1* to positively regulate transcription [36]. A separate study showed that CDK1-meditated phosphorylation of UHRF1 results in release from the deubiquitylase USP7 and subsequent proteasomal degradation during M phase [37]. As our data and other published data sets have shown that UHRF1 mRNA is decreased in multiple senescent contexts, we speculate that UHRF1 downregulation is mainly because of the sequester of E2F1 by hypo-phosphorylated retinoblastoma protein (RB), which has been commonly observed in senescent cells. This mechanism may also explain the dynamic UHRF1 expression in basal cells after airway injury, which is tightly correlated with cell proliferation.

In conclusion, our study comprehensively outlines the mechanistic basis of UHRF1-mediated senescence suppression, which consists of both proper assembly of the replication machinery as well as maintenance of epigenetic patterns subsequent to DNA synthesis. As this epigenetic signaling network inhibits the senescent cell fate in mammalian stem cell populations, it could have important implications for diseases broadly related to stem cell dysfunction and the ageing process.

## Materials and Methods

### Animals

*Uhrf1flox/flox* (*Uhrf1 fl/fl*) mice were kindly provided by Dr Haruhiko Koseki in the RIKEN Center for Integrative Medical Sciences. In *Uhrf1*^*fl/fl*^ mice, exon 4 and exon 5 were flanked by *loxP* sites [17]. *K5-CreER* mice were kindly provided by Dr Brigid Hogan in the Department of Cell Biology at Duke University, Durham, NC, USA. The CreERT2 fragment, driven by the internal ribosome entry site, was placed into the 3′-untranslated region of *Krt5* gene [38]. All mice were maintained on a C57BL/6 background. To induce *Uhrf1* deletion in basal cells, male mice 8- to 12-week old were intraperitoneally injected with four doses of Tmx (75 μg g^−1^ of body weight) every other day. One week after the final dose, mice were exposed to 500 p.p.m. of SO_2_ in air for 4 h. All experiments were approved by the Duke Institutional Animal Care and Use Committee.

### Cell culture

Primary HBE cells were obtained from dead healthy donors under a University of North Carolina Biomedical Institutional Review Board-approved protocol (03-1396). P0 cells were cultured in plastic dishes coated with bovine collagen in basal epithelial growth medium (Lonza, Walkersville, MD, USA). Confluent P0 cells were subsequently passaged into dishes without collagen and frozen down for experiments. For replicative senescent cells, HBE cells were kept passaging for 80 days when HBE cells stopped growing and had elevated SA-β-gal activity. Human diploid fibroblasts IMR90 cells were cultured in Eagle's minimum essential medium supplemented with 10% fetal bovine serum and 1×penicillin streptomycin. 293T cells were maintained in Dulbecco’s modified Eagle’s medium supplemented with 10% fetal bovine serum.

For bronchosphere culture, spheres were cultured in 0.4-μm pore size polyester membranes in 24-well, 6.5-mm Transwell inserts (Corning, Tewksbury, MA, USA). In all, 100 μl of 100% Matrigel (BD Biosciences, San Jose, CA, USA) was first added to the apical surface of an insert as a cushion. In all, 1 200 HBE cells mixed with 24 000 MRC-5 (ATCC, CCL-171) human lung fibroblasts in 100 μl of 50% ALI/50% Matrigel were added on the top of the cushion. ALI media were added to the lower chamber and changed every other day. The pan-caspase inhibitor Z-VAD-FMK was added to the ALI media and changed every other day for 7 days. The concentration of this compound was 10 μM. For immunohistochemistry, spheres were fixed with 4% (wt/vol) paraformaldehyde (PFA) in phosphate-buffered saline (PBS) for 30 min at room temperature and then embedded in paraffin. Three independent experiments were performed using HBE cells from three different donors. Images were taken by EVOS microscopy, Thermo Scientific, Asheville, NC, USA at indicated time points.

For ALI culture, the same inserts were used and coated with collagen IV (C7521; Sigma-Aldrich, St Louis, MO, USA). In all, 33 000 cells were seeded into inserts. ALI media were applied in both lower chamber and inserts. When the culture became confluent 1 week after seeding, media were removed from inserts and left dry. For whole-mount staining, ALI cultures were washed with PBS for three times and fixed in 4% (wt/vol) PFA in room temperature for 10 min.

### Plasmid and lentivirus

The control shRNA and the shRNA constructs targeting UHRF1 were transduced into cells using PLKO.1-puro lentivirual vector. All shRNA constructs were purchased from Sigma-Aldrich. The guide sequences of the shRNAs used in this study are as follows: shU_1, 5′ -CGTCATTTACCACGTGAAATA-3′; shU_2, 5′- CCGCACCAAGGAATGTACCAT-3′. For the CRISPR/Cas9-mediated gene knockout experiment, the lentiCRISPR v2 construct from Dr Feng Zhang’s lab was purchased from Addgene (Cambridge, MA, USA) [39]. UHRF1 sgRNA_1: 5′- CGCCGACACCATGTGGATCC-3′; UHRF1 sgRNA_2, 5′ -ACACCATGTGGATCCAGGTT-3′. To overexpress H-Ras to induce senescence, H-Ras V12 was cloned into a Tet-on inducible plasmid. In all, 12 μg of individual shRNA, lentiCRISPR v2 or the tet-on construct, 9 μg of ps-PAX_2_ and 3 μg of vesicular stomatitis virus G glycoprotein (VSV-G) expressing plasmids were co-transfected into 293T cells using Lipofectamine 2000 (Thermo Fisher Scientific, Asheville, NC, USA) based on the manual. Viruses were collected 48 h after transfection. For HBE cells, when cells reached 40% confluency, viruses were applied for 8 h with 2 μg ml^−1^ polybrene. For IMR90 cell virus transduction, cells were infected with viruses for 18 h at 40% confluency with 4 μg ml^−1^ polybrene. Forty-eight hours after virus transduction, stable UHRF1 knockdown cells were selected using puromycin (0.5 μg ml^−1^ for HBE cells; 1 μg ml^−1^ for IMR90 cells) for 2 days. For the tet-on system, IMR90 fibroblasts were infected with viruses for 18 h at 40% confluency with 4 μg ml^−1^ polybrene. After 3-day puromycin selection, the final concentration of 1 μg ml^−1^ Dox was added for 8 days to induce senescence, and cells were collected for subsequent experiments.

### EdU staining

Cells were incubated with 10 μM EdU for 2 h at 37 °C. Spheres were incubated with 10 μM EdU for 5 h at 37 °C. (EdU staining kit, Thermo Fisher Scientific) was performed using either Click-iT EdU flow cytometry assay kit or Click-iT EdU imaging kit according to the manufacturer’s protocol.

### Annexin V and PI staining

Cells were harvested and immediately stained with Alexa Fluor 488 Annexin V/Dead Cell Apoptosis Kit (Thermo Fisher Scientific). Flow cytometry was performed in the Duke Cancer Center shared flow cytometry facility.

### Cell cycle analysis

Cells were incubated with 10 μM EdU for 2 h and then fixed on 4 °C for 1 h by using the Foxp3/Transcription Factor Fixation Permeabilization Concentrate and Diluent buffer (eBioscience, catalogue number: 00-5521-00, Thermo Scientific Fisher). Subsequently, cells were stained with Click-iT EdU flow cytometry kit and treated with RNases before staining with PI for 30 min in room temperature.

### Western blot

Total protein was extracted using 0.9% NP-40, 1mM EDTA, 50mM Tris-HCl, 50mM NaCl (NETN) buffer supplemented with protease inhibitor cocktails (Thermo Fisher Scientific). Lysates were first sonicated and centrifuged at 12 000 r.p.m. for 10 min. Supernatant was collected and boiled in loading buffer for 10 min. In all, –30 μg protein samples were loaded on denaturing sodium dodecyl sulfate gels and transferred to nitrocellulose membranes (Millipore, Billerica, MA, USA). Membranes were blocked in 5% bovine serum albumin tris-buffered saline, 0.1% Tween 20 (TBST) and probed with following antibodies: primary antibody anti-UHRF1 (BD, San Jose, CA, USA, 612264, 1:1 000), p21 (Cell Signaling, Danvers, MA, USA, 2947, 1:1 000), p15 (Santa Cruz, Dallas, TX, USA, sc-612, 1:1 000), p16 (BD, 51-1325GR, 1:1 000), p53 (Santa Cruz, sc-126, 1:1 000), secondary antibody labeled by horseradish peroxidase (Invitrogen). The secondary antibody was visualized using a chemiluminescent reagent Pierce ECL kit (Thermo Fisher Scientific).

### Immunofluorescence and immunohistochemistry

For PCNA staining, HBE cells or IMR90 cells transduced with either scramble or UHRF1-targeting shRNA were fixed in ice-cold methanol for 5 min in room temperature. Cells were blocked with serum-free buffer (DAKO, Carpinteria, CA, USA) with 0.1% Triton X-100 and incubated with the following antibodies: primary anti-PCNA (Santa Cruz, sc-7907, 1:200), anti-UHRF1 (Santa Cruz, sc-373750, 1:100) and secondary antibody conjugated with different Alexa fluorophores (Invitrogen). Mouse tracheas were fixed in 4% (wt/vol) paraformaldehyde in PBS at 4°C for 3 h, washed with PBS and paraffin embedded. Tracheas were sectioned longitudinally at 7 μm. For the antigen retrieval step, paraffin sections were soaked in 10-mM sodium citrate butter, pH 6, in a pressure cooker-like device (Biocare Medical, Pacheco, CA, USA). For ALI whole-mount staining, membranes were stripped off from the inserts and fixed in 4% paraformaldehyde for 10 min in room temperature. Antibodies included anti-UHRF1 (Santa Cruz, sc-373750, 1:50), anti-KRT5 (Covance, Denver, CO, USA, 1:1 000), anti-KRT8 (DSHB, Iowa City, IO, USA, TROMA-I, 1:200), anti-Ki67 (Abcam, Cambridge, MA, USA, ab15580, 1:1 000), anti-PCNA (Santa Cruz, sc-7907, 1:50), MUC5A (Lab Vision, Fremont, CA, USA, MS145-PO,1:200), acetylated tubulin (Sigma; T-7451, 1:1 000). Alexa Fluor-labeled secondary antibodies (Invitrogen) were used at a 1:400 dilution. All immunofluorescent images were recorded by Zeiss 780 confocal upright fixed stage confocal microscope with 20×/0.80 Dry Zeiss Plan-Apochromat objective or 63×/1.4 Oil Zeiss Plan-Apochromat objective at room temperature. The acquisitions were performed with Zen software (Carl Zeiss, Dublin, CA, USA). Subsequent images were rotated, cropped and contrast adjusted using the ImageJ software. The Imaris software was used for three-dimensional reconstruction.

### SA-β-gal assays

SA-β-gal assay was performed as previously described [21]. For the cytochemical assay, control or UHRF1 knockdown HBE cells or IMR90 cells were washed twice in PBS and then fixed using 2% formaldehyde and 0.2% glutaraldehyde in PBS for 5 min at room temperature. Cells were subsequently stated with 40 mM citric acid/sodium phosphate buffer (pH 6.0), 5 mM potassium ferrocyanide, 5 mM potassium ferracyanide, 150 mM NaCl, 2 mM MgCl_2_ and 1 mg ml^−1^ X-Gal, typically 8-h overnight at 37 °C. Microscopic analyses were performed using an Olympus CK40 microscope (Center Valley, PA, USA) with DP20 camera. Three fields were counted for each group. Experiments were repeated three times using cells from three different donors. For the fluorescence-activated cell sorting-based sorting of senescent cells, we added C12FDG, a substrate that produces a fluorescent product upon cleavage by SA-β-gal, to the culture medium and subsequently sorted out different cells populations based on fluorescent intensity [21].

### DNA methylation analysis by bisulfite sequencing

Genomic DNA was extracted and purified with DNA Isolation kit (Qiagen, Santa Clarita, CA, USA). In all, 1 μg genomic DNA was used for bisulfite conversion using the MethylDetector Kit (Active Motif, Carlsbad, CA, USA). All procedures were based on the manufacturer’s instruction. The converted DNA was then amplified by PCR using EpiMark Hot Start Taq DNA Polymerase (New England, Ipswich, MA, USA). Bisulfite sequencing primers are designed based on Methprimer online software [40]. Forward PCR primer: 5′- GTTTGGATTGTTTTTGGGAAAAAG-3′; reverse PCR primer: 5′- CCTAACATCTTTAAACAAACTTCCCC-3′. Subsequently, the PCR products were subcloned into pCR 2.1-TOPO vector (Invitrogen) according to the manual. Five clones were picked up for each experimental group and sent out for sequencing.

### RNA-sequencing and gene ontology analysis

RNAs from control and UHRF1 knockdown groups were extracted by RNeasy Mini Kit (Qiagen). RNA-seq library preparation, sequencing and analysis were done by Beijing Genomics Institute (Shenzhen, Guangdong, China). Differentially expressed genes were estimated by using NOIseq method [41]. Genes with probability ⩾0.8 and fold change >2 between control and knockdown groups were considered significantly changed. For gene ontology analysis, to find more genes that are changed in both shRNAs, we lowered probability to 0.7 and found 89 downregulated genes and 84 upregulated genes. Those genes were then subjected to PANTHER Classification System [42].

### Statistical analysis

Unless otherwise indicated, Student’s *t*-tests were used for all statistical analyses. Data are reported as mean±s.e.m.
