# Supplementary material for: UHRF1 is required for basal stem cell proliferation in response to airway injury
Source: Cell Discov. 2017 Jun 13;3:17019–. doi: 10.1038/celldisc.2017.19 (PMC5468773; doi:10.1038/celldisc.2017.19)
Supplement: Supplementary information [file celldisc201719-s1.pdf]

Supplementary Figures
Figure S1

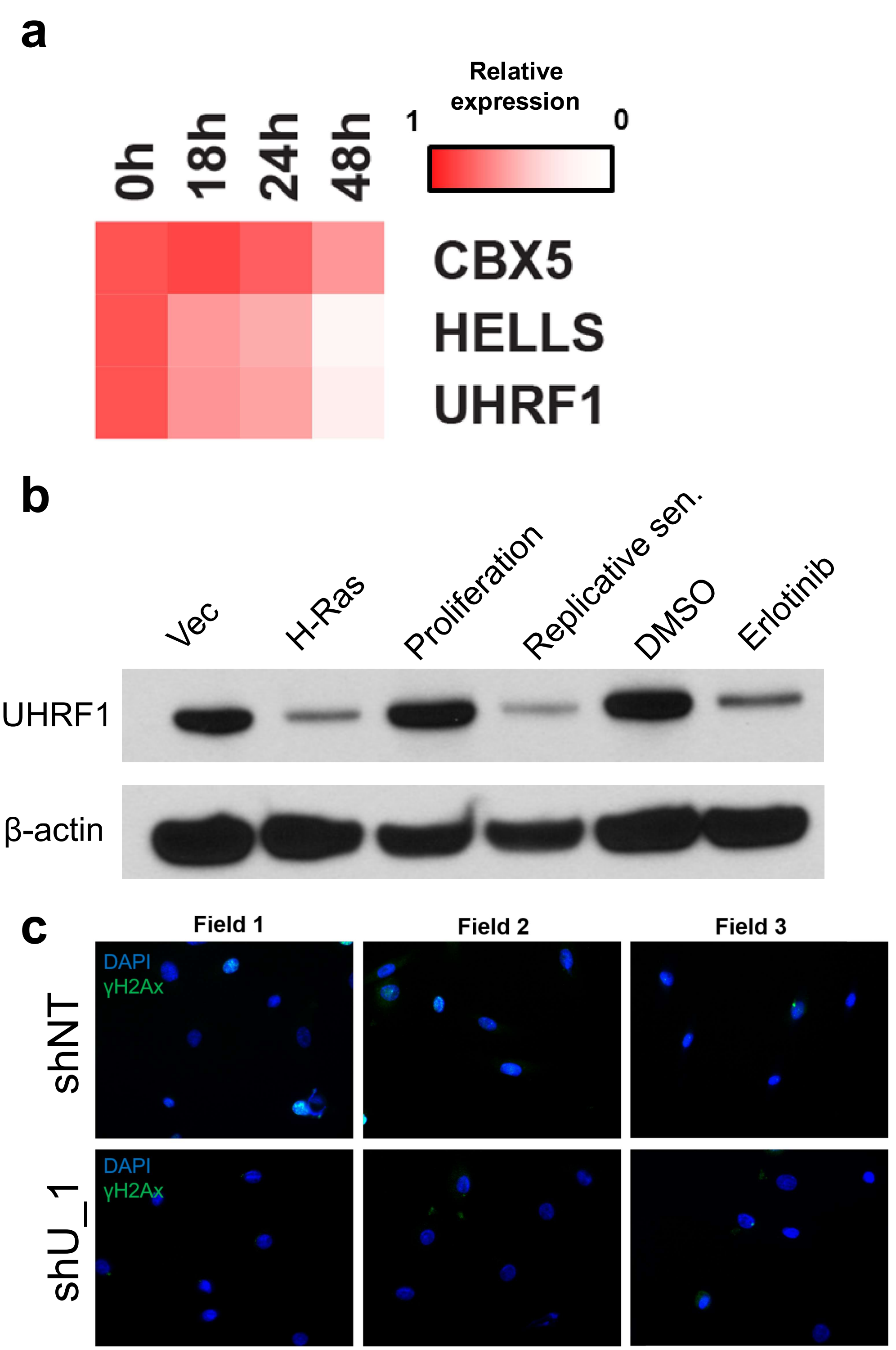

Figure S1 (**a**) Time-course q-PCR validation of the three epigenetic regulators. Cells were treated with 1uM erlotinib, and the heat map represents the fold change of each epigenetic modifier. (**b**) Whole cell lysates from indicated groups were collected and subsequently immunoblotted with UHRF1 and β-actin antibodies. For oncogenic H-Ras V12 overexpression, samples were collected from IMR90 cells after ten days-doxycycline treatment to induce H-Ras V12 expression. Most cells in the H-Ras V12 group were senescent. Replicative senescent HBE cells were cultured for fourty days when cells ceased proliferating. For Erlotinib treatment-induced senescence, HBE cells were treated with either DMSO as control or 1uM Erlotinib for three days to induce senescence. Replicative sen.: replicative senescent. (**c**) Control or UHRF1-knockdown senescent HBE cells were fixed with 2% paraformaldehyde and stained with the γH2Ax antibody.

Figure S2

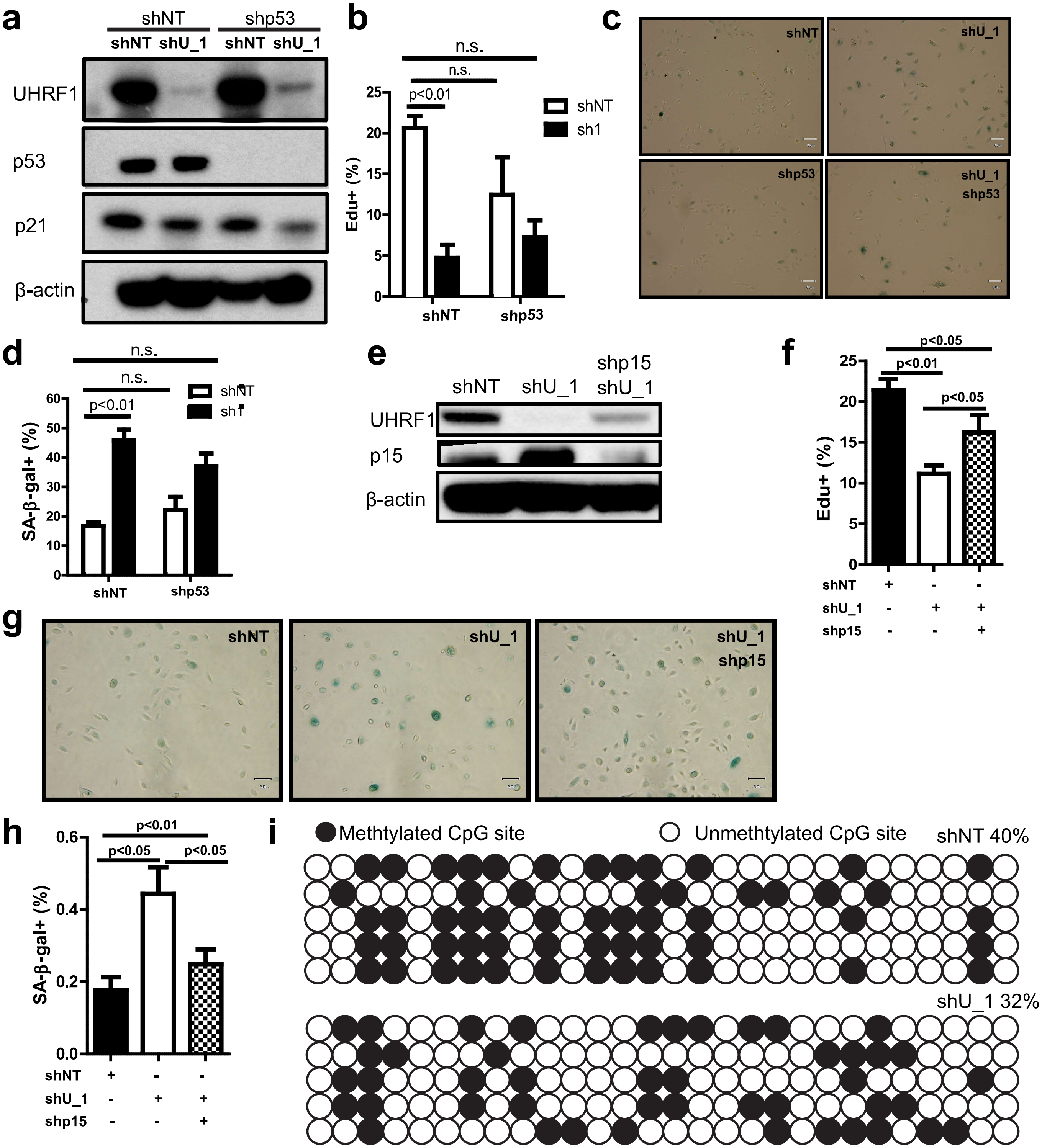

Figure S2 The proliferation defect and senescent phenotype of UHRF1-deficient HBE cells partially relies on the p15/INK4B pathway. (**a**) Immunoblot results of p53 knockdown in UHRF1 knockdown cells, and protein levels of both UHRF1 and p53 were successfully reduced. (**b**) Cell proliferation was measured in control cells (shNT), UHRF1 knockdown cells, p53 knockdown cells or p53 and UHRF1 co-knockdown cells by EdU incorporation. Quantified data was shown as mean percentage ± SEM values (n=3). Statistical significance was determined by two-way ANOVA (F=3.81, no statistical significance; post hoc t-test of shNT and shU_1, $p<0.01$). (**c**, **d**) Upper panels show the result of SA-βgal staining and quantification is shown in (d). Quantified data is shown as mean percentage ± SEM values (n=3). Statistical significance was determined by two-way ANOVA (F=3.68, no statistical significance; post hoc t-test of shNT and shU_1, $p<0.01$). (**e**) Immunoblot results of p15 knockdown in UHRF1 knockdown cells. Both UHRF1 and p15 proteins were successfully reduced. (**f**) Cell proliferation was measured in control cells (shNT), UHRF1 knockdown cells and UHRF1 and p15 co-knockdown cells by EdU incorporation. (**g**, **h**) Upper panels show the result of SA-βgal staining and quantification is shown in (h). Data are reported as mean ± SEM. (**i**) Bisulfite sequencing of the CpG island in the promoter region of p15. 28 CpG sites were analyzed. Filled circles represent methylated cytosine whereas unfilled circles represent unmethylated cytosine.

Figure S3

GSE 69058

Gene.symbol	Gene.title	logFC	P.Value
Ppbp	pro-platelet basic protein	8.49	9.28E-11
Il33	interleukin 33	5.71	1.33E-07
Fst	follistatin	4.94	2.97E-06
Hells	helicase, lymphoid specific	4.75	1.25E-08
2810417H13Rik	RIKEN cDNA 2810417H13 gene	4.5	2.16E-09
Fst	follistatin	4.5	1.08E-06
Lgals1	lectin, galactose binding, soluble 1	4.48	2.82E-07
Rrm2	ribonucleotide reductase M2	4.23	7.44E-10
Fabp5	fatty acid binding protein 5, epidermal	4.21	3.18E-09
Lgals1	lectin, galactose binding, soluble 1	4.19	3.70E-07
Mtm1	X-linked myotubular myopathy gene 1	4.08	4.68E-08
Uhrf1	ubiquitin-like, containing PHD and RING finger domains, 1	4.07	1.17E-08
Sprr2a2///Sprr2a	small proline-rich protein 2A2///small proline-rich protein 2A1	4.03	1.21E-10
Gm5593///Ccnb1	predicted gene 5593///cyclin B1	4.03	2.32E-08
Ncapg	non-SMC condensin I complex, subunit G	4.01	7.53E-09
Cdca5	cell division cycle associated 5	3.99	1.32E-08
Lrr1	leucine rich repeat protein 1	3.99	1.97E-07
Cxcl3	chemokine (C-X-C motif) ligand 3	3.99	1.29E-06
Rrm2	ribonucleotide reductase M2	3.95	1.94E-08
Gm5593///Ccnb1	predicted gene 5593///cyclin B1	3.92	4.91E-10

Figure S3 The dataset GSE69058 was reanalyzed. Highly changed expression genes from control and SO_2 injured airway epithelium were sorted by logFC. *Uhrf1* was the twelfth highest upregulated gene in tracheal epithelial cells following injury.

Figure S4

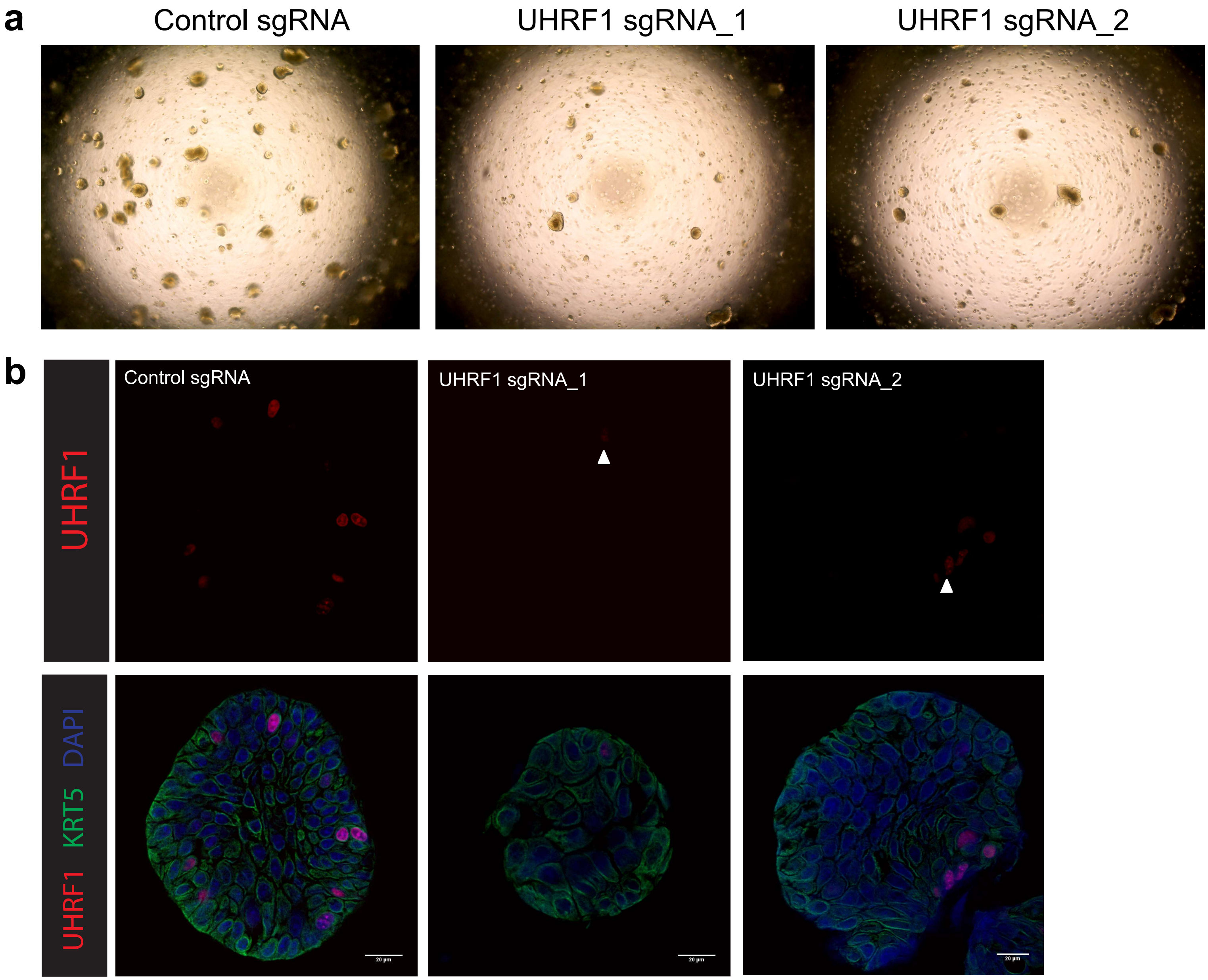

Figure S4 UHRF1 knockout in HBE cells using CRISPR/Cas9 abrogates the sphere formation capacity. (**a**) Representative DIC microscopy images of spheres at day 7 from HBEs. (**b**) Confocal images of individual spheres from control and UHRF1 knockout groups stained with indicated antibodies. Spheres formed in the knockout groups likely result from incomplete deletion of UHRF1. Scale bar: 20 um.

Figure S5

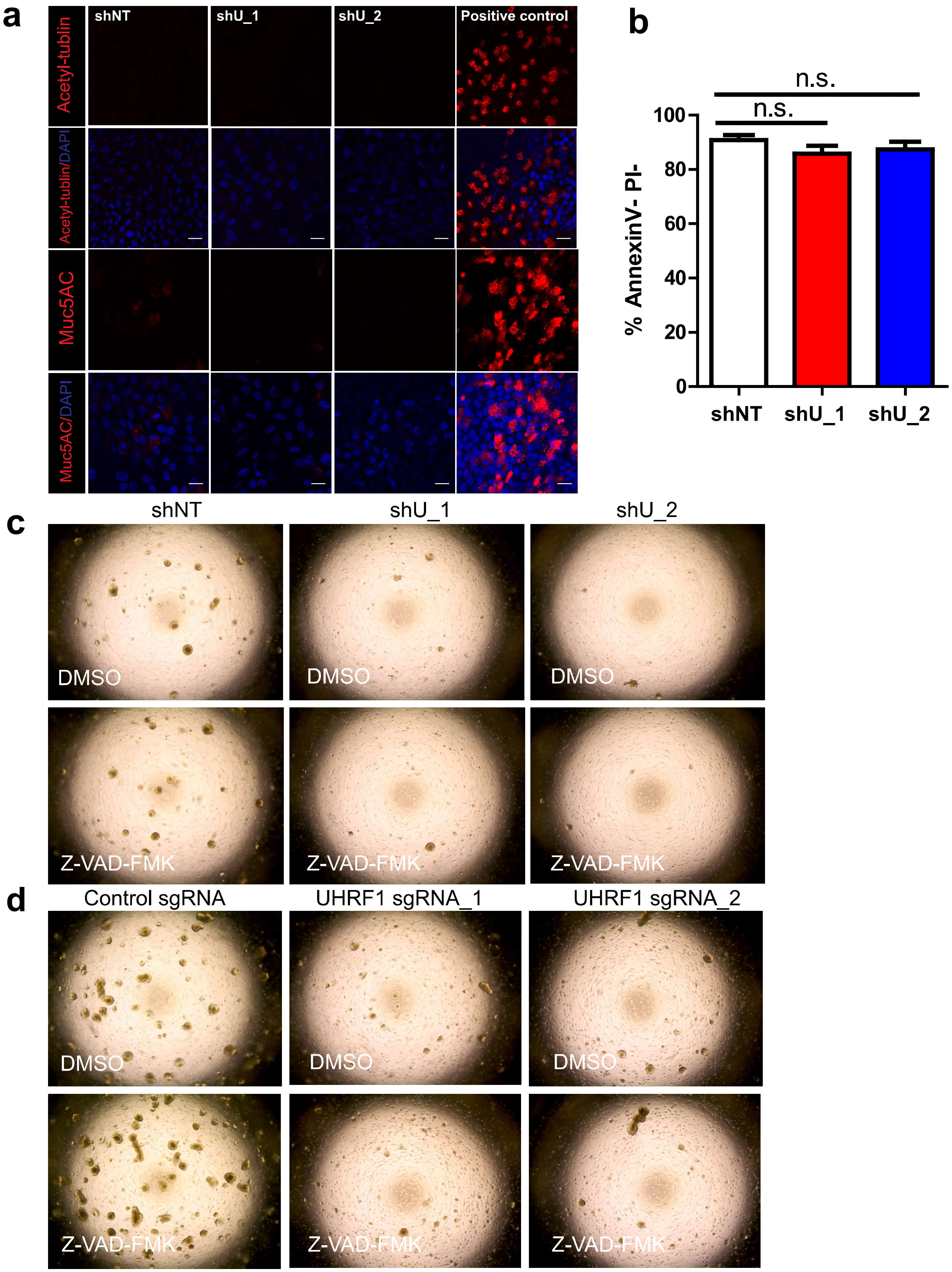

Figure S5 UHRF1 knockdown in HBE cells does not affect cell differentiation or cell viability. (**a**) Representative confocal images of the whole-mount ALI cultures from control cells (shNT) or UHRF1-knockdown cells (shU_1 and shU_2). Muc5Ac marks goblet cells, and Acetyl-tublin marks ciliated cells. Positive controls were collected from the same batch of unmanipulated HBE cells one month after plating, suggesting the success of staining and differentiation. Scale bar: 20 um. (**b**) The result of Annexin V and PI staining of control and UHRF1-knockdown HBE cells. No statistical significance of percentages of Annexin V-PI cells was found between groups. (**c**, **d**) Representative DIC microscopy images of spheres at day 7 from HBE cells. Control or UHRF1-deficient HBE cells were treated with 10uM pan-caspase inhibitor Z-VAD-FMK for 7 days when cells were plated.

Figure S6

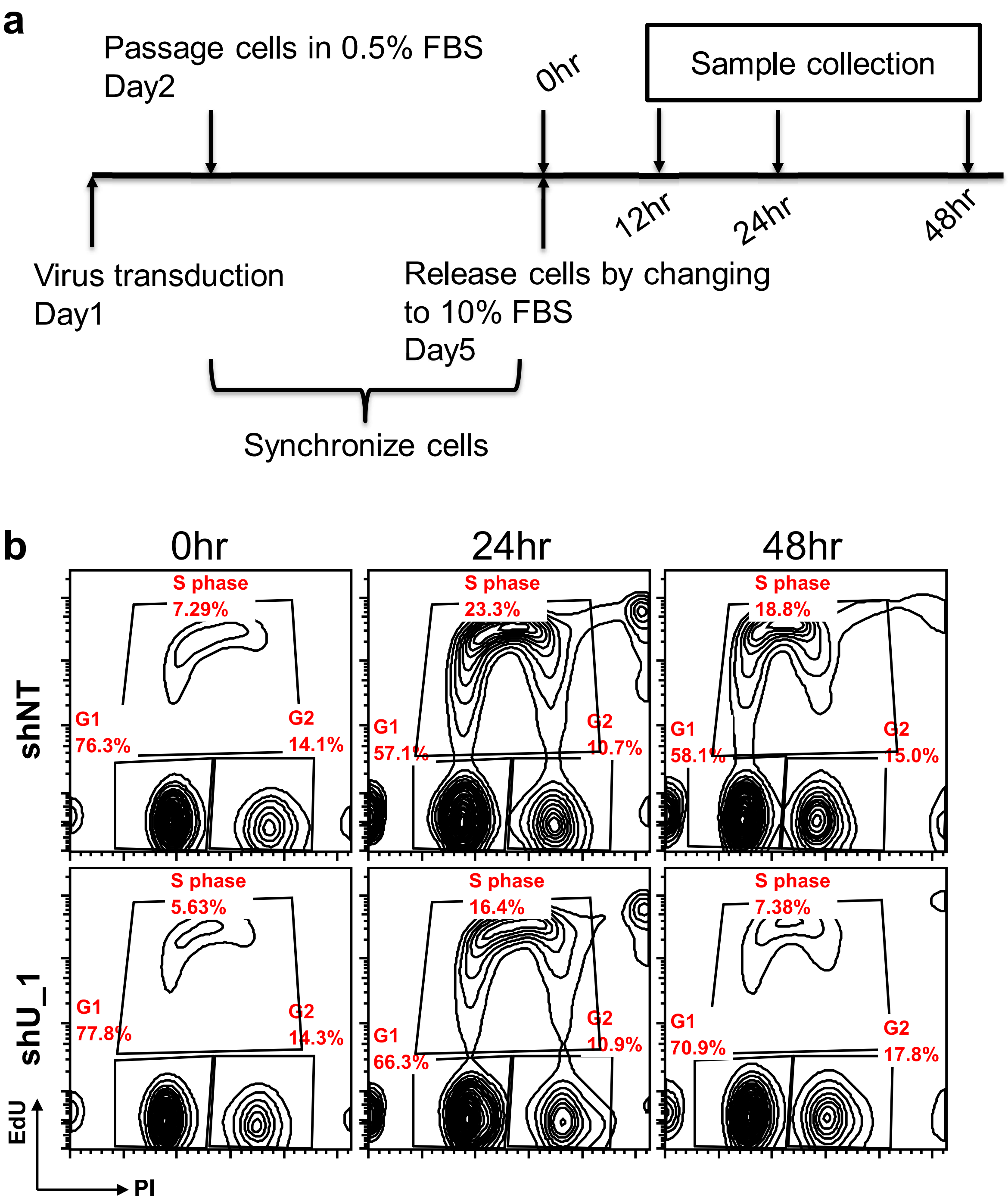

Figure S6 (**a**) Illustration of the experimental procedure. IMR90 cells were transduced with control or shUHRF1 viruses and then synchronized in G1 phase by culturing in 0.5% serum. Cells were then released by addition of serum. Samples were collected at the indicated time points. (**b**) Cell cycle profile was determined by PI and EdU double staining.

Figure S7

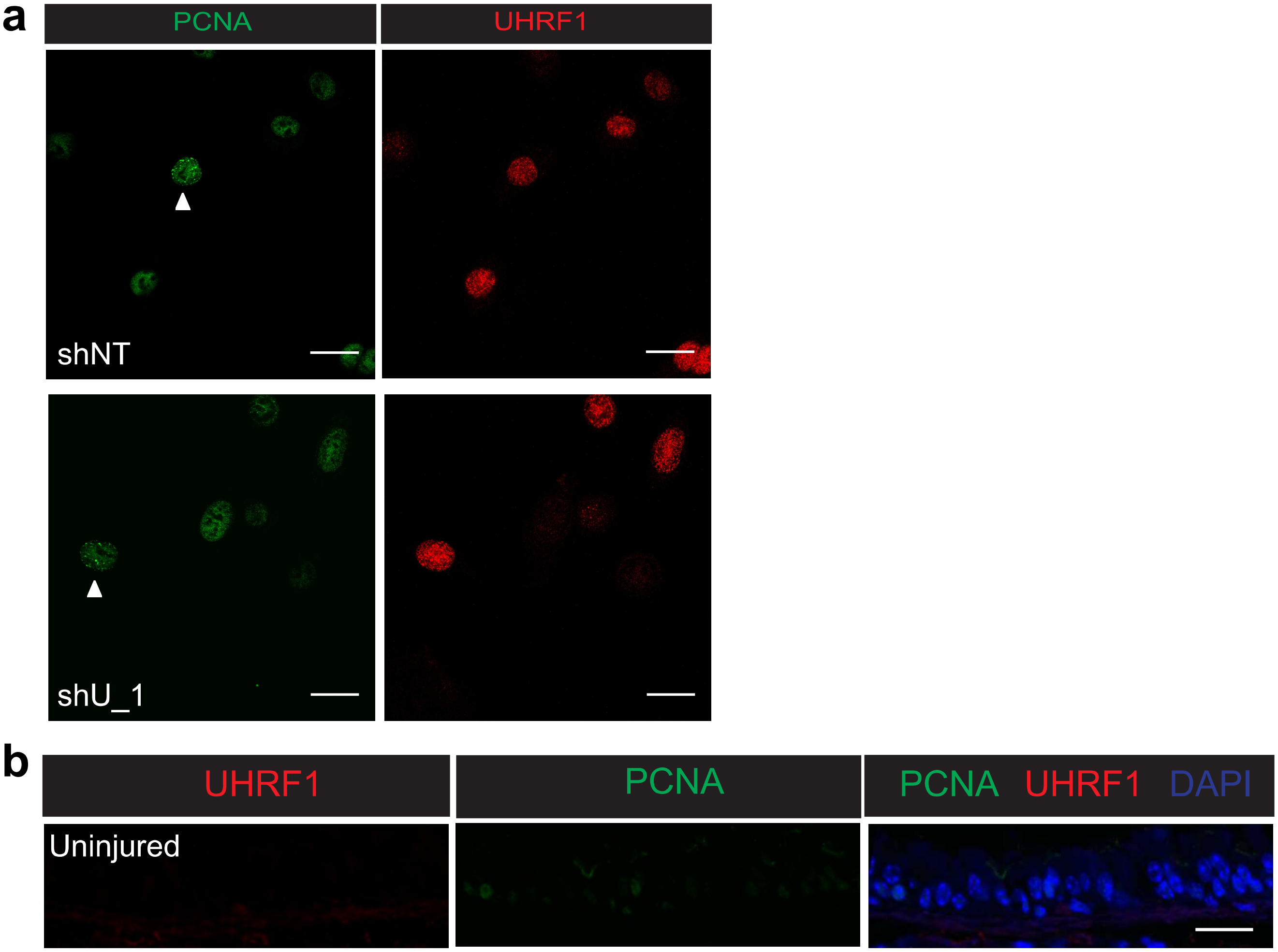

Figure S7 PCNA staining pattern in UHRF1 knockdown HBE cells and in steady-state airway epithelium. (**a**) Representative confocal images of control and UHRF1 knockdown cells co-stained with indicated antibodies. Cells were collected 2 days after virus transduction when UHRF1 level remained intact. (**b**) Representative confocal images of uninjured airway epithelium stained with indicated antibodies. Scale bar: 20 um
